# Circ_0004851 regulates the molecular mechanism of miR-296-3p/FGF11 in the influence of high iodine on PTC

**DOI:** 10.1186/s12967-024-05405-2

**Published:** 2024-06-20

**Authors:** Jing-jing Li, Zi-xuan Ru, Xu Yang, Jing-xue Sun, Yan-mei-zhi Wu, Xiao-yao Yang, Bo-yu Hou, Bing Xue, Chao Ding, Hong Qiao

**Affiliations:** 1 Department of Endocrinology and Metabolism, The Second Affiliated Hospital of Harbin Medical University, Harbin, 150086 Heilongjiang China; 2 Department of Science and Education, Heilongjiang Provincial Hospital, Harbin, 150036 Heilongjiang China; 3 Department of General surgery, The Second Affiliated Hospital of Harbin Medical University, Harbin, 150086 Heilongjiang China; 4 NHC Key Laboratory of Etiology and Epidemiology, Harbin Medical University, No. 157 Baojian Road, Nangang District, Harbin, 150081 Heilongjiang China

**Keywords:** Iodine, PTC, Circ_0004851, miR-296-3p, FGF11, Tumor development

## Abstract

The prevalence of papillary thyroid cancer (PTC) has been rising in recent years. Despite its relatively low mortality, PTC frequently metastasizes to lymph nodes and often recurs, posing significant health and economic burdens. The role of iodine in the pathogenesis and advancement of thyroid cancer remains poorly understood. Circular RNAs (circRNAs) are recognized to function as competing endogenous RNAs (ceRNAs) that modulate gene expression and play a role in various cancer stages. Consequently, this research aimed to elucidate the mechanism by which circRNA influences the impact of iodine on PTC. Our research indicates that high iodine levels can exacerbate the malignancy of PTC via the circ_0004851/miR-296-3p/FGF11 axis. These insights into iodine’s biological role in PTC and the association of circRNA with the disease could pave the way for novel biomarkers and potentially effective therapeutic strategies to mitigate PTC progression.

**Supplementary Information:**

The online version contains supplementary material available at 10.1186/s12967-024-05405-2.

## Introduction

On a global scale, thyroid cancer (TC) is recognized as the most prevalent endocrine malignancy. Recent decades have seen a steady incline in TC incidence [1], with papillary thyroid cancer (PTC)—prevalent in iodine-sufficient regions like China—constituting over 70% of these cases [2]. PTC, typically highly differentiated, boasts a post-5-year survival rate exceeding 90% [3]. However, recurrence occurs in 10-15% of PTC cases, significantly impacting survival and life quality for these patients.

Iodine significantly influences PTC development, with its role in thyroid cell proliferation and differentiation being well-documented. Abnormal iodine levels, either deficient or excessive, are implicated in thyroid pathology [4]. Epidemiological evidence suggests a higher PTC incidence in iodine-deficient than in iodine-excess regions, indicating iodine’s critical role in thyroid cancer progression [5]. Furthermore, the *BRAF* V600E gene mutation is the most common genetic mutation in PTC, with an average incidence of about 45% [6]. Interestingly, previous studies have concluded that among the iodine metabolism genes, *BRAF* V600E is a highly specific target for PTC [7]. Nonetheless, the impact of high iodine levels on existing thyroid cancer and its underlying mechanisms warrant further investigation.

Non-coding RNAs (ncRNAs) is identified as pivotal in tumorigenesis, encompassing various forms such as transfer RNAs, ribosomal RNAs, long ncRNAs (lncRNAs), small ncRNAs—including microRNAs, piRNAs, snoRNAs, snRNAs, exRNAs—and circular RNAs (circRNAs) [8]. CircRNAs, a novel class of ncRNAs, are gaining prominence as clinical biomarkers due to their differential expression in tumors and associations with diagnosis and prognosis [9, 10]. Their regulatory role over cancer cell phenotypes—such as proliferation, apoptosis, and migration—underscores their significance in tumor genesis and progression [11]. These molecules are instrumental in post-transcriptional regulation and protein synthesis via miRNA interaction [12–14]. In thyroid cancer, high iodine has been shown to foster tumor growth by down-regulating mir-422 A and up-regulating MAPK1 [15]. Excess iodine has also been observed to elevate Smad4 levels, consequently inhibiting PTC proliferation by suppressing miR-146b-5p expression [16]. Varied KIO_3_ doses exhibit dichotomous effects on thyroid cancer cell malignancy, potentially through mir-146a regulation and downstream gene expression [17]. Given that circRNAs possess miRNA binding sites, they can modulate miRNA activity, attenuating the miRNA-mediated inhibition of target genes, thereby influencing mRNA production [8]. The prospect of circRNAs competitively inhibiting miRNAs, thus affecting mRNA expression in thyroid cancer, presents a novel mechanism by which iodine could influence the disease.

This research aims to delineate the association involving serum iodine levels and PTC characteristics, including tissue location, size, number, and lymph node metastasis. We observed high iodine’s effect on PTC cell biology. Utilizing high-throughput sequencing, we identified circRNA-miRNA-target gene interactions modulated by iodine excess in PTC patients. This facilitated the elucidation of the underlying mechanisms in iodine-influenced PTC, leading to the construction of a circ_0004851/miR-296-3p/FGF11-regulated signaling network. Additionally, high-iodine PTC mouse models were developed to monitor tumor progression.

## Materials as well as methods

### Clinical case collection

From October 2019 to October 2022, patients who underwent thyroid surgery at the Second Affiliated Hospital of Harbin Medical University and received a postoperative pathological confirmation of benign thyroid nodules, or PTC, were included in this research. Medical records were meticulously compiled, comprising patient identifiers and general information such as gender, age, and body mass index, along with pathological details like cancer typology and stage. The selection criteria mandated: [1] no familial thyroid disease history; [2] abstention from iodine-containing medications or supplements for the preceding year; [3] avoidance of iodine contrast agents in procedures (e.g., coronary angiography, ERCP, enhanced CT) within the last six months, and no intake of amiodarone or contraceptives; [4] exclusion of pregnant individuals, patients with diabetes, or renal complications; and [5] a three-day pre-sampling abstinence from iodine-rich foods or supplements. Serum was extracted from a 5 ml sample of fasting venous blood and stored in six hours. The preservation of serum and fresh tissues occurred at -80 °C pending extraction and assessment. Staging adhered to the eighth edition of the AJCC TNM system for thyroid cancer. Informed consent, detailing the research’s objectives and methodology, was obtained from each participant, ensuring compliance with the Declaration of Helsinki. The Medical Ethics Committee of Harbin Medical University granted approval for this research.

### Serum iodine

Serum iodine concentration was measured using cerium arsenate-catalysed spectrophotometry, aligning with the national health industry standard W/T572-2017 [18]. Classification of serum iodine levels was conducted in accordance with WHO recommendations [https://www.who.int/publications/i/item/9789241595827]: categorizing as < 45 µg/L, 45–90 µg/L, and > 90 µg/L.

### Cell lines and cultures

The human PTC cell line BCPAP was sourced from Wuhan Punosai, the TPC-1 PTC cell line from the Chinese Academy of Sciences cell bank, and the Nthy-ori-3-1 normal thyroid cell line out of Zhongqiao Xinzhou. Authentication procedures were carried out for all cell lines. The culture medium comprised 90% RPMI 1640, 10% FBS, and 1% penicillin-streptomycin. Cultivation occurred in a controlled environment maintained at 37 °C with 5% CO_2_.

### Cell proliferation assay

Cells were placed at a density of 5,000 cells per well into 96-well plates and incubated overnight. They were then treated with various reagents in a complete medium. The assessment of proliferative capacity was conducted in accordance with the manufacturer’s instructions utilizing the Cell Counting Kit-8 (CCK-8, Dojindo).

### Assay of wound healing

A total of 5 × 10^5^ cells were cultivated per well in 6-well plates. Upon the cell monolayer had attained 60% confluence, a scrape was incised using a sterile pipette tip. Following a scrubbing with PBS, cells were incubated with 1% FBS in RPMI 1640 medium containing varying iodine concentrations. Microscopic images were captured at baseline and after 24 h to assess migration, which was quantified using ImageJ software. This assay was replicated a minimum of three times per condition.

### Transwell invasion assay

Transwell inserts (Corning) were coated with a matrix gel solution (BD Biosciences) at a 1:8 dilution and allowed to set for 60 min at 37 °C. Cell suspensions under various conditions were placed into the inserts, and the lower chambers were filled with medium to which 20% FBS was added. After a 24-hour incubation at 37 °C, cells on the membrane were fixed with methanol and stained with 0.1% crystal violet. Invasion was observed with an inverted microscope (Olympus), and cells were counted. Each experimental condition was conducted in triplicate.

### Flow cytometry assessment

Apoptotic cells were detected using the Annexin V FITC/PI Apoptosis Detection Kit (BD, US). The collected cells were washed with cold PBS and processed per the kit’s instructions. assessment was conducted using a BD Accuri C6 Plus flow cytometer, with FlowJo V10 software employed to enumerate live, early apoptotic, as well as late apoptotic cells.

### High-throughput RNA sequencing

RNA-Seq technology was used to identify differentially expressed circRNAs, miRNAs, and mRNAs in PTC tissues from patients with high iodine levels and those with normal iodine levels. This was achieved using high-throughput sequencing and ceRNA screening criteria. Candidates were selected based on a |log2FoldChange| greater than 1 and a *p*-value of less than 0.05 for genes of interest.

### RT-qPCR validation

RT-qPCR, employing SYBR Green for detection, was utilized to confirm the expression of circRNAs, miRNAs, and mRNAs identified through high-throughput sequencing. Trizol reagent was used for total RNA extraction, followed by cDNA synthesis. RT-qPCR was conducted with SYBR® Premix Ex Taq. Internal reference genes GAPDH and U6 were employed for circRNAs/mRNAs and miRNAs, respectively. The primer sequences employed for tissue and cellular validation are detailed in Table S1. Expression levels of circRNAs, miRNAs, and mRNAs were computed, with RT-qPCR data analyzed using the 2^−ΔΔCT^ method for relative quantification.

### Western blot

Western blotting was carried out to determine FGF11, PARP, Pro-Caspase and Cleaved-Caspase protein levels, using GAPDH as a loading control. Tissue and cellular proteins from PTC samples were lysed with RIPA buffer containing PMSF, and concentrations were normalized using the BCA Protein Assay. Following SDS-PAGE, proteins were transferred to PVDF membranes and incubated with primary antibodies at 4 °C overnight, then with secondary antibodies (111-035-003, 115-035-003, Jackson) at room temperature for 1 h. Visualization was achieved via an image gel analyzer and ECL detection system, with band intensity quantified using ImageJ software. All Western blot procedures were replicated three times. The primary antibodies used in this study included: FGF11 (DF8944, Affinity), PARP (13371-1-AP, proteintech), Caspase (19677-1-AP, proteintech), GAPDH (60004-1-Ig, proteintech).

### Sanger sequencing

For sequencing assessment, five samples at a single locus were examined using one primer pair. Primer design was facilitated by Primer3 online software (https://bioinfo.ut.ee/primer3-0.4.0/). Following purification with Promega’s Shrimp Alkaline Phosphatase as well as Epicentre’s Exonuclease I, the PCR products were sequenced employing the BigDye3.1 kit from ABI. The sequencing reactions were conducted on an ABI3730 following alcohol purification.

### RNA fluorescence in situ hybridisation (FISH)

A Cy3-labeled probe targeting circ_0004851 was designed and synthesized by Genepharma. DAPI staining was employed to visualize cell nuclei. Sample assessment was conducted using a Nikon inverted fluorescence microscope. RNA FISH was carried out strictly adhering to the manufacturer’s protocol provided by Genepharma.

### Dual luciferase reporter assay

To ascertain direct RNA interactions, luciferase reporter assays were employed. Constructs of the circ_0004851 wild-type (WT) 3′-UTR as well as FGF11 WT 3′-UTR, along with their mutant counterparts, circ_0004851 (MUT) 3′-UTR as well as FGF11 MUT 3′-UTR, were synthesized. Following seeding HEK293T cells into 24-well plates, they were co-transfected with 600 ng each of the WT or mutant plasmids of circ_0004851 and FGF11, along with 600 ng of miR-296-3p mimics or negative controls. Fluorescence intensity was measured 48 h post-transfection using the Dual-Luciferase Reporter Assay System (Promega, Madison, USA), following the manufacturer’s protocol.

### Construction of Si-circ_0004851, MicroRNA mimics and inhibitors, and FGF11 overexpression vector

Circ_0004851 knockdown was achieved through transient RNA interference. Si-circ_0004851, microRNA mimics, inhibitors, and the FGF11 overexpression plasmid were supplied by Genepharma Pharmaceuticals Technology Co., Ltd. (Shanghai, China). The sequences of the siRNA, microRNA mimics, and inhibitors are displayed in Table S2.

### Transplantation tumor model in nude mice

BALB/c-nude female mice were housed in IVC cages within a specific pathogen-free (SPF) facility. Randomly divided into two groups: the BCPAP normal group (iodine concentration < 10 µg/L in deionized water, *n* = 5), and the BCPAP high iodine group (iodine concentration at 6000 µg/L contained with KIO_3_ in deionized water, *n* = 5), each mouse received an injection of 50 × 10^6^ cells. Tumor volume was calculated as 0.5 × (length × width^2^). Tumor volume and body weight were measured every three days. Post-tumor development, the mice were euthanized for PCR and WB assessment of the tumors. All animal procedures complied with Harbin Medical University’s guidelines for animal care and were approved by the Animal Care and Use Committee.

### Statistical assessment

Upon verifying the raw data’s integrity, SPSS version 22.0 and Graphpad Prism 5 were utilized for statistical assessment as well as graphical data representation, respectively. Measured data are presented as mean ± standard deviation for conformity to normal distribution and as median (M) for non-conformity to normal distribution, while count data are presented as frequency (%). Two-group comparisons were conducted using the t-test, multiple group comparisons with ANOVA, and pairwise comparisons with the Tukey test. Percentages (rates) between groups were assessed using the Chi-square (X^2^) test. Depending on the variable’s nature (binary or continuous), logistic and linear regression analyses were conducted for multifactorial analysis. Pearson’s assessment was applied for correlation evaluations. A *p*-value < 0.05 denoted statistical significance (*: p less than 0.05, **: p less than 0.01, ***: p less than 0.001).

## Results

### High iodine was associated with PTC tumor location, size, number and lymph node metastasis, promoting PTC cell proliferation, migration as well as invasion and inhibiting apoptosis

The research enrolled 1,431 participants adhering to the predefined inclusion and exclusion criteria. This cohort included 1,178 individuals diagnosed with PTC, with a gender distribution of 20.5% male and 79.5% female, and a benign nodule cohort comprising 253 individuals, with 20.9% male and 79.1% female. The mean age with standard deviation for the PTC and benign nodule groups was 45.47 ± 10.27 years (range 18–73 years) and 54.21 ± 11.18 years (range 24–80 years), respectively. The average body mass index was 25.14 ± 4.427 kg/m^2^ for the PTC group and 20.44 ± 9.784 kg/m^2^ for the benign group. The median serum iodine levels were 60.32 µg/L in the PTC group and 62.41 µg/L in the benign nodule group, both within the acceptable range for iodine. The clinical and demographic features of the research population are detailed in Table 1, including sample size, gender, age, BMI, and serum iodine concentration.

**Table 1:** Basic information of studied samples

	PTC group	Benign group
Male	242(20.5%)	53(20.9%)
Female	936(79.5%)	200(79.5%)
Age(years)	45.47 ± 10.27	54.21 ± 11.18
BMI(kg/m2)	25.14 ± 4.427	20.44 ± 9.784
SIC(µg/L)	60.32	62.41

The research population’s iodine nutritional status was categorized into three groups following the serum iodine stratification criteria set by the World Health Organization (WHO). This classification aided in examining the association between serum iodine levels and the clinicopathological characteristics of Papillary Thyroid Carcinoma (PTC). A significant correlation was found between serum iodine and the presence of cancer in one or both lobes of the thyroid, the size of the tumor, the number of tumors present, and the incidence of lymph node metastasis in PTC cases. However, serum iodine levels did not show a significant relationship with the infiltration of surrounding thyroid tissue or the TNM stage of PTC (refer to Table 2).

**Table 2:** Association between serum Iodine and clinicopathological characteristics among patients with papillary thyroid carcinoma

Characteristic	*N*	SIC(ug/L)*n* (%)			X^2^	*p*
		< 45	45–90	> 90		
Thyroid lesions						
Bilateral	313	41	228	44	8.289	**0.016**
Unilateral	853	129	652	72		
Tumor size(cm)						
< 1	807	121	624	62	13.251	**0.001**
≥ 1	325	45	232	48		
Tumor number						
Single	750	111	580	59	9.252	**0.01**
Multiple	410	59	296	55		
Tissue infiltration						
positive	578	77	437	64	2.886	0.236
Negative	584	95	436	53		
Lymph node involvement						
positive	498	71	365	62	6.282	**0.043**
Negative	662	100	509	53		
TNM stage						
I/II	1062	153	806	103	2.704	0.259
III/IV	110	19	76	15		

**Fig. 1:**
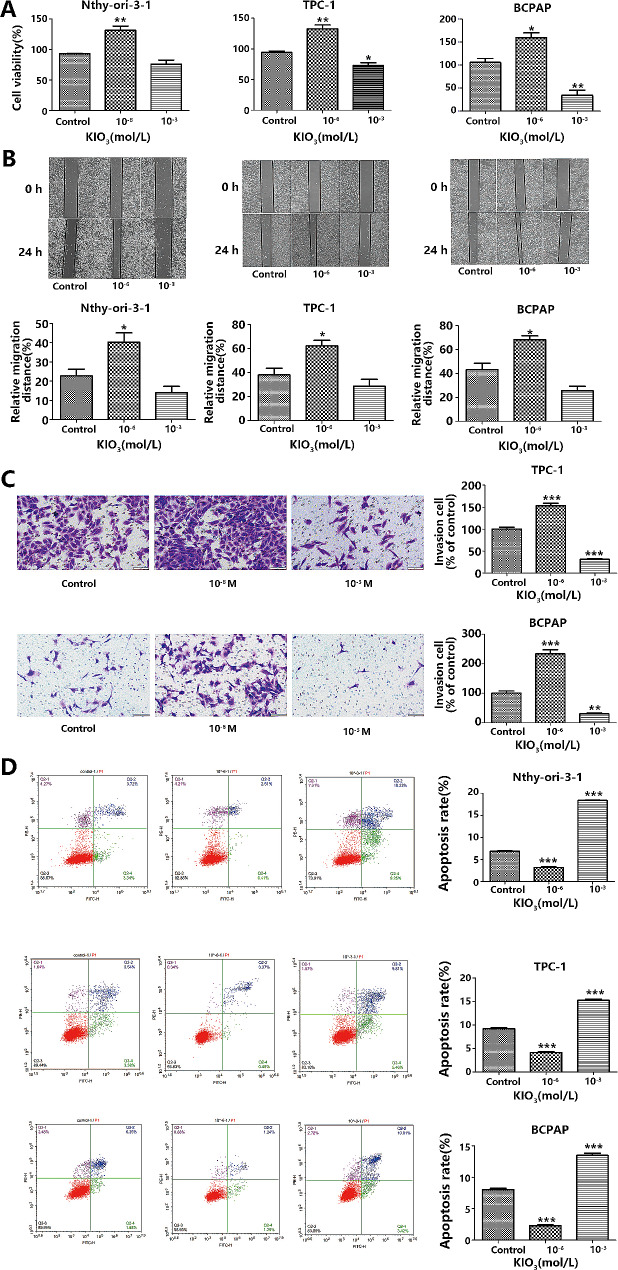
Effect of Iodine on cell proliferation, migration, invasion and apoptosis. **A**. CCK8 verified Nthy-0ri-3-1、TPC-1 and BCPAP cell viability at different Iodine concentrations; **(B)** The scratch wounds and the migration cells in the scratch were photographed at 0 h and 24 h after scratching; **(C)** Cell invasion was detected by Transwell invasion assay. **(D)** Apoptosis was examined using Annexin V/pI staining followed by flow cytometry, total apoptotic cells are the sum of early apoptotic cells and late apoptotic cells. *p < 0.05, **p < 0.01, ***p < 0.001 (vs. the control group)

Our prior experimental findings indicated that iodine impacts BCPAP cell proliferation in a biphasic manner: initially increasing to a peak at 10^− 6^ mol/L, followed by a decline to a nadir at 10^− 3^ mol/L where it notably inhibited cell growth. Consequently, we selected three potassium iodate concentrations for this research: control (0 mol/L), 10^− 6^ mol/L, and 10^− 3^ mol/L. We compared the proliferation rates at 24 h, 48 h, and 72 h across these concentrations and observed no significant temporal correlation, thereby justifying the selection of a 24-hour interval for subsequent experiments [19]. To investigate iodine’s impact on PTC cells, we utilized a CCK-8 kit, wound healing assay, flow cytometry, and Transwell assays to assess the proliferation, migration, invasion, and apoptosis of Nthy-0ri-3-1, TPC-1, and BCPAP cell lines under various KIO_3_ concentrations (0, 10^− 6^ mol/L, 10^− 3^ mol/L). The outcomes showed heightened sensitivity to 10^− 6^ mol/L KIO_3_, significantly increasing proliferative (Fig. 1A), migratory (Fig. 1B), invasive (Fig. 1C), and reduced apoptotic capabilities (Fig. 1D). In contrast, the response to 10^− 3^ mol/L KIO_3_ was adverse. The biological behavior of PTC cells at 10^− 6^ mol/L KIO_3_ paralleled the effects of high iodine levels observed in PTC patient cohorts; thus, we adopted 10^− 6^ mol/L KIO_3_ to mimic high iodine exposure in vivo for our subsequent experimental procedures.

### Construction of circRNA-Mediated ceRNA networks under iodine exposure

**Fig. 2:**
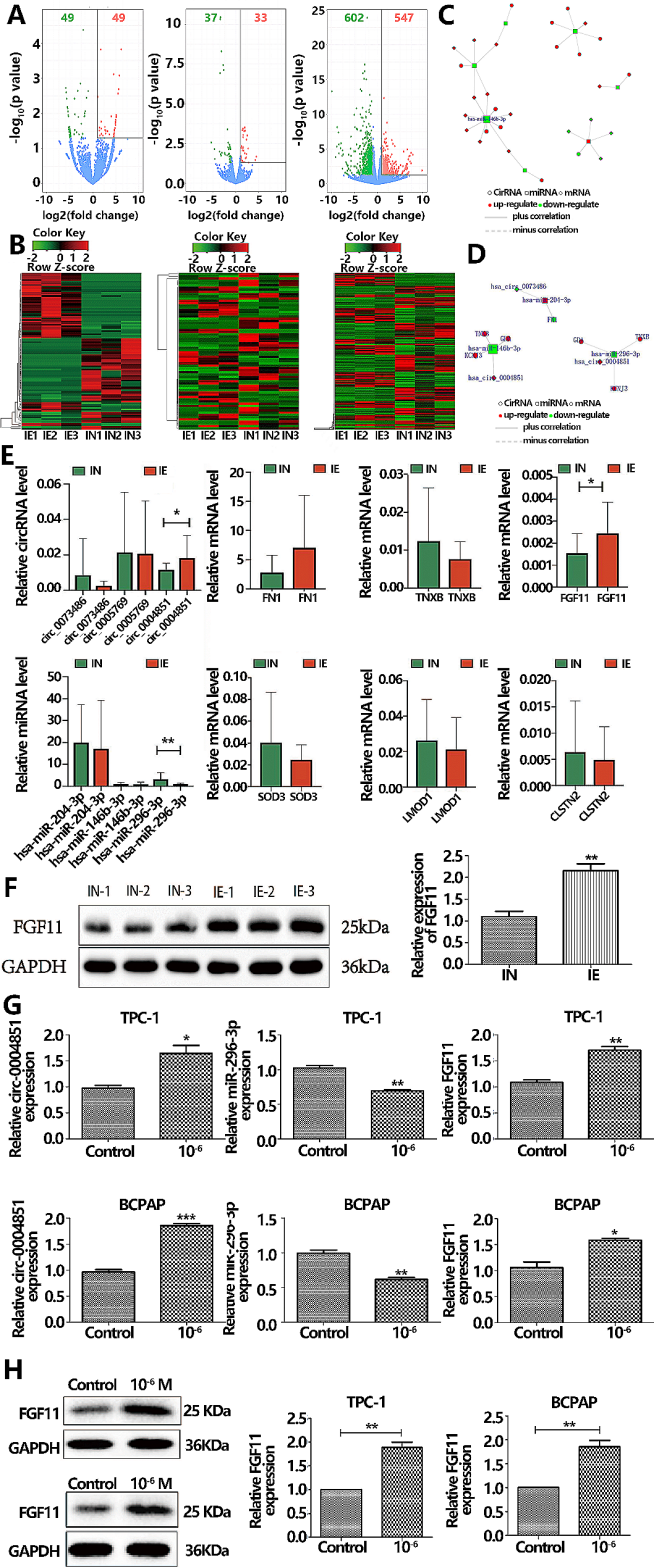
Construction of ceRNA network mediated by circRNA under iodine exposure. **A**. Volcano map. Red points represent up-regulated genes. Green points represent down-regulated genes. Blue points represent insignificant differentially expressed genes. **B**. Significant difference clustering heat map. Red color represents high gene expression in the sample, while green color represents low gene expression in the sample compared to the IN group. **C**. A total of 38 circRNA/miRNA/mRNA relationships were identified, including 7 miRNAs, 16 circRNAs and 13 mRNAs. **D**. Six ceRNA axes were identified for validation. the red indicates the up-regulated gene and the green indicates the down-regulated gene. **E**. Reverse transcription-quantitative PCR was used to detect the expression levels of 3 miRNAs, 3 circRNAs and 6 mRNAs in PTC tissues. **F**. The expression level of FGF11 protein was detected by Western blot in PTC tissues. **G**. PCR for circ_0004851、miR-296-3p、FGF11 in PTC cell. **H**. Western blot for FGF11 in PTC cell

Aligning with WHO guidelines, serum iodine levels were classified as < 45 µg/L, 45–90 µg/L, and > 90 µg/L. Selection for whole transcriptome sequencing was based on a dietary survey. Three cases each of high-iodine tissues (IE) and iodine-suitable tissues (IN) were chosen for sequencing, taking dietary surveys and inclusion/exclusion criteria into account. Transcriptome assessment identified expression patterns of circRNAs, miRNAs, and mRNAs in PTC tissues. Notably, 98 circRNAs were differentially expressed in high-iodine thyroid cancer tissues versus the iodine-suitable group (log2|fold change| ≥ 1, p less than 0.05), with an equal number being up-regulated and down-regulated. A selection of 88 microRNAs met the differential expression criteria (log2|fold change| ≥ 1, p less than 0.05), comprising 40 up-regulated and 48 down-regulated genes. Moreover, 1149 mRNAs were differentially expressed (log2|fold change| ≥ 1, p less than 0.05), including 547 up-regulated and 602 down-regulated genes (illustrated in Fig. 2A: Volcano Plot and Fig. 2B: Heat Map).

In accordance with the competitive endogenous RNA (ceRNA) hypothesis, we constructed a circRNA-miRNA-mRNA network via bioinformatics assessment. This network comprises 38 ceRNA axes, involving 16 circRNAs, 7 microRNAs, and 13 mRNAs (depicted in Fig. 2C). Of these, we selected 7 ceRNA axes based on their FPKM values (gene expression metric log2(FPKM + 1)), and upon consultation with the Genecards database, identified ADH6 as having minimal expression in thyroid tissue. Consequently, we confirmed the tissue expression of 6 remaining ceRNA axes, excluding ADH6 (illustrated in Fig. 2D). Notably, miR-296-3p within this network drew our focus due to its link to PTC progression. To validate the accuracy of differentially expressed genes identified by high-throughput sequencing, qRT-PCR was employed. We measured the expression levels of circRNA, miRNA, and mRNA across six ceRNA axes in 22 high-iodine PTC tissue samples and 26 iodine-adapted PTC tissue samples, corroborating the RNA sequencing data. The expression pattern of the has-circ_0004851/miR-296-3p/FGF11 axis in high iodine samples was in agreement with the transcriptome sequencing outcomes when compared to the control group (shown in Fig. 2E). Western blot assessment of PTC specimens further confirmed the elevated expression of FGF11 in the high-iodine cohort (Fig. 2F). Validation experiments at control and 10^-6 mol/L potassium iodate concentrations demonstrated that the expression trends of has-circ-0004851, miR-296-3p, and FGF11 were consistent with the findings at 10^-6 mol/L potassium iodate and that FGF11 protein levels aligned with the PCR data (Fig. 2G-H). These outcomes indicate a significant upregulation of FGF11 gene expression in response to high iodine exposure, and its presence across various malignant tumors implies that FGF11 could serve as a pivotal function in iodine-induced PTC development. Thus, we have prioritized the circ_0004851/miR-296-3p/FGF11 axis for further investigation.

### Validation of circ-0004851 localization and the circ-0004851/miR-296-3p/FGF11 axis

**Fig. 3:**
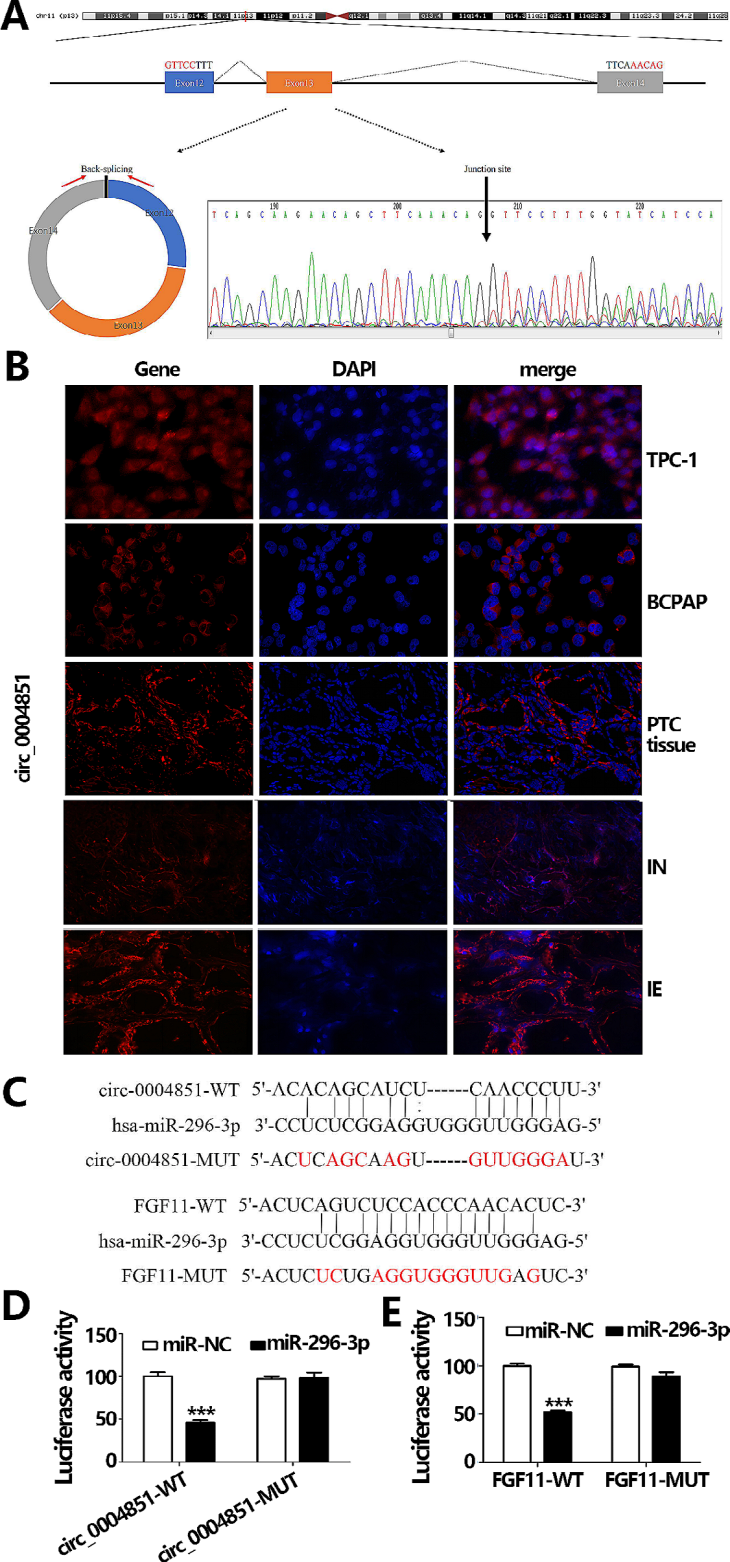
Verify the real existence and localization of circRNA, miR-296-3p targets circ_0004851 and FGF11. **A.** The diagram illustrates the divergent primers and Sanger sequencing of purified PCR products, revealing the backsplice junction sequences of the mentioned circRNAs. The backsplice site is marked by a black arrowhead. **B.** Circ_0004851 displayed cytoplasmic localization as observed by Fluorescence in situ hybridization (FISH). **C**. FISH observed Circ_0004851 levels in tissue samples with different iodine concentrations. **(D)** The conjunction points of miRNA-296-3p on circ_0004851 and FGF11. **(E)** Luciferase reporter-gene activity of miR-296-3p co-transfection with circ_0004851 WT and MUT, co-transfection with FGF11 WT and MUT. Data are shown as means ± SD of three replicate experiments. *p < 0.05, **p < 0.01, ***p < 0.001 versus mimic NC

Sanger sequencing confirmed the head-to-tail splice junction in the circ_0004851 qRT-PCR product, expectedly sized at 412 base pairs (bp). Utilizing specifically designed divergent primers, we aligned the Sanger sequencing outcomes with the CircBase sequence, verifying the existence of circ_0004851 (Fig. 3A). Fluorescence in situ hybridization (FISH) demonstrated circ_0004851’s cytoplasmic localization (Fig. 3B and C), suggesting that it may be involved in the development of PTC at the post-transcriptional level. Besides, the FISH results also showed that the expression level of circ_0004851 was higher in the IE group compared with IN group (Fig. 3B). The in vitro targeting relationship was further elucidated using dual-luciferase reporter assays.

Figure 3D and E show a significant reduction in fluorescence activity of the wild-type recombinant plasmid upon miR-296-3p mimic interference in each group, confirming the interaction between miR-296-3p and the binding sites on the wild-type circ_0004851 and FGF11 recombinant plasmids (p less than 0.05). These findings collectively establish the interactive association involving miR-296-3p, circ_0004851, and FGF11.

### Inhibition of Circ_0004851 reverses iodine-induced functional changes in PTC cells

**Fig. 4:**
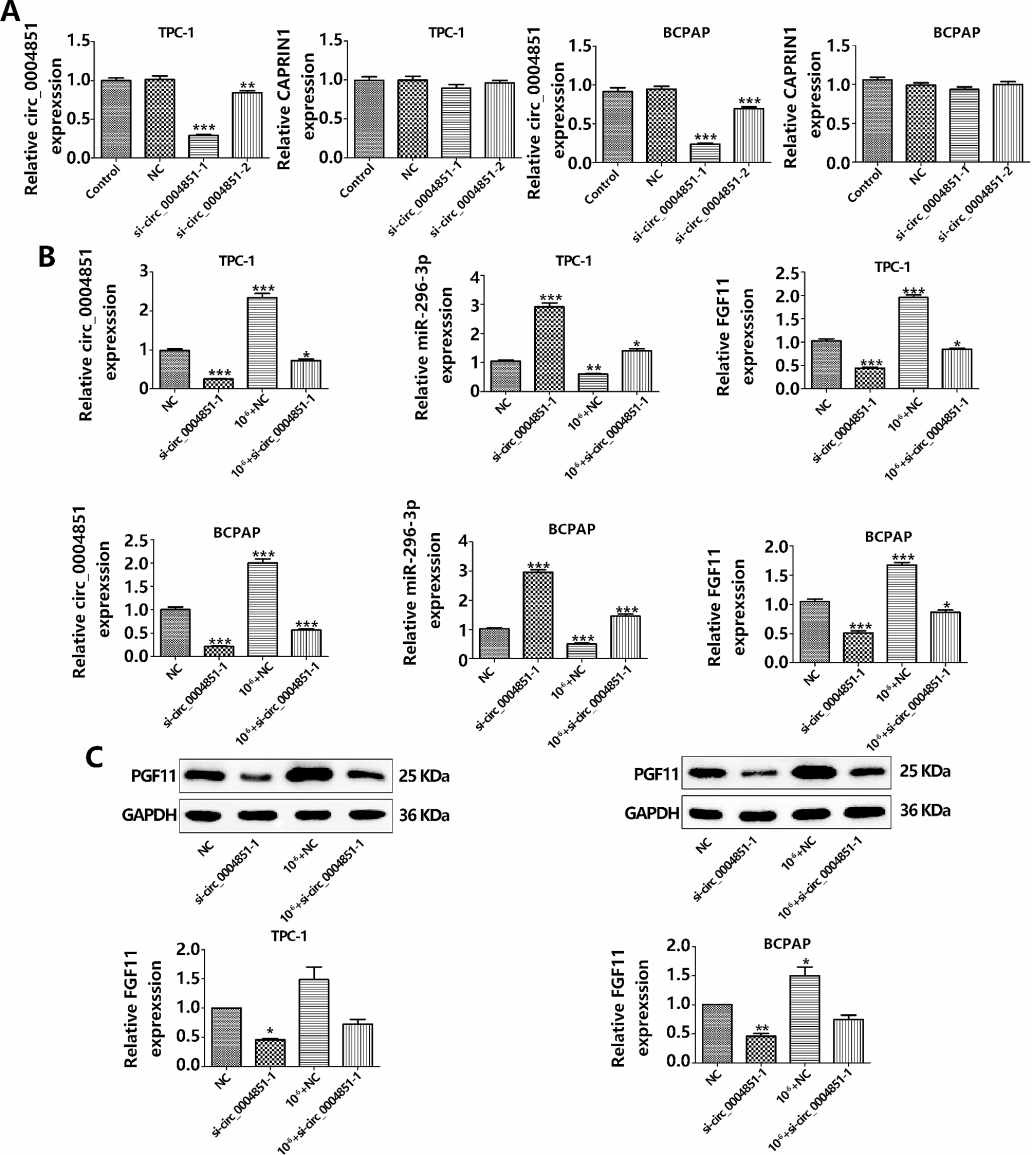
The expression level of circ_0004851, miR-296-3p and FGF11 under silencing circ_0004851. **A**.TPC-1 and BCPAP cells were stably transfected with circ_0004851 short hairpin RNAs or the vector plasmid, and the expression levels of circ_0004851 and CAPRIN1 mRNA were detected by real-time PCR. **B**. Circ_0004851 was silenced and the expression of circ_0004851, miR-296-3p and FGF11 was detected by PCR. **C.** Silencing circ_0004851, Western blot for FGF11 in PTC cell

**Fig. 5:**
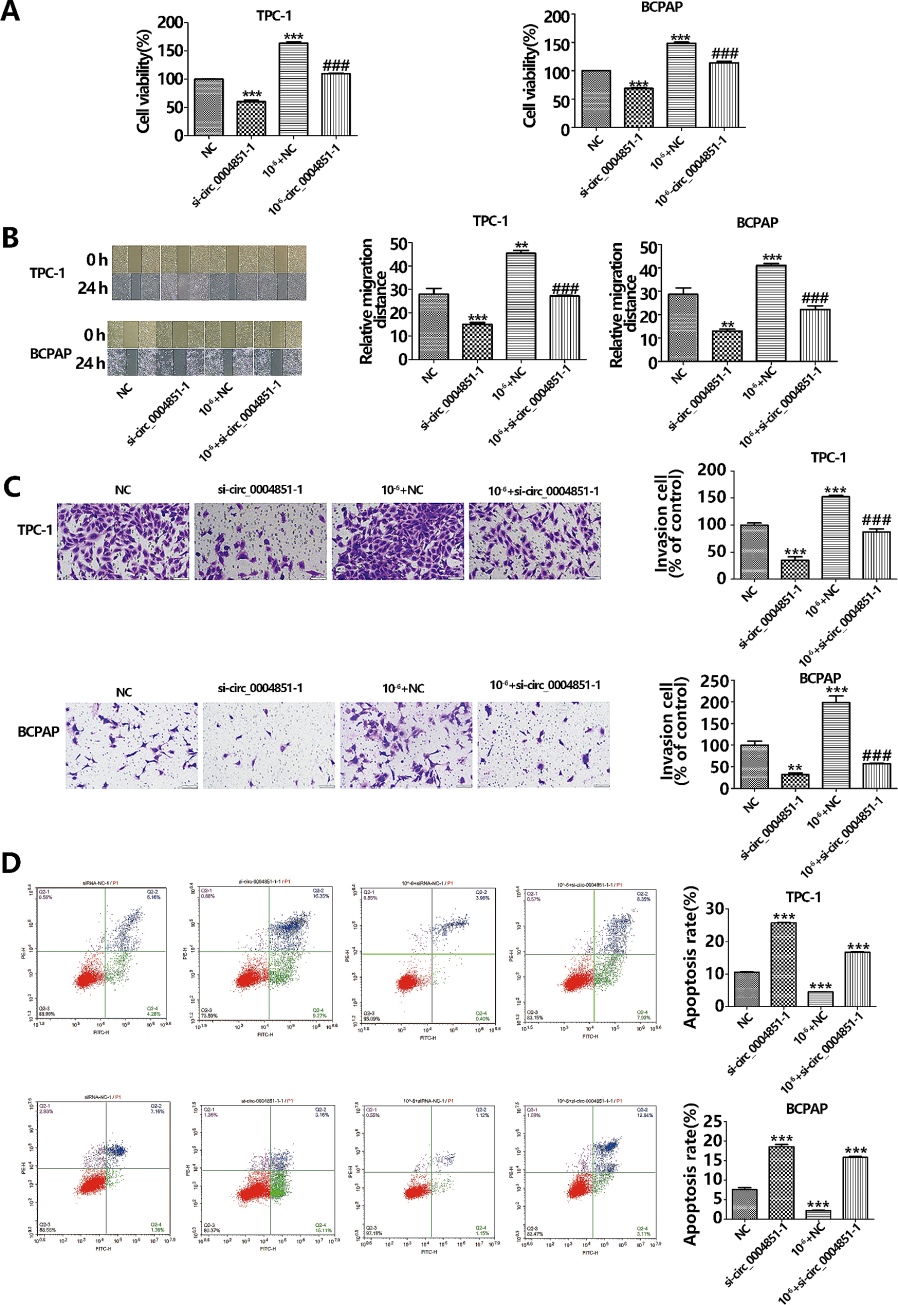
Circ_0004851 silencing inhibits the proliferation migration, invasion, and promotes apoptosis of PTC cells. **A**. Proliferation of TPC-1 and BCPAP cells transfected with si-circ_0004851 was measured by CCK-8 assay. **B.** The effect of si-circ_0004851 on migration was evaluated by the wound-healing assay in TPC-1 and BCPAP cells. **C.** Transwell Matrigel invasion assays were conducted to evaluate the invasion abilities of TPC-1 and BCPAP cells transfected with si-circ_0004851 or vector. **D**. Apoptosis of TPC-1 and BCPAP cells transfected with si-circ_0004851 was measured flow cytometry

Circ_0004851 originates from the CAPRIN1 gene’s exons 12–14 and remains unexplored in thyroid cancer research. We investigated its function within PTC cells by transfecting TPC-1 and BCPAP cells with specially designed siRNAs targeting circ_0004851, which suppressed its expression, confirmed by qRT-PCR. Notably, si-circ_0004851-1 significantly reduced circ_0004851 levels without affecting CAPRIN1 mRNA (Fig. 4A). Further experiments involved transfecting TPC-1 and BCPAP cells with si-circ_0004851-1 or si-NC under iodine and non-iodine conditions, followed by evaluating mRNA and protein levels of circ_0004851, miR-296-3p, and FGF11. Circ_0004851 knockdown diminished circ_0004851 and FGF11 expression while enhancing miR-296-3p levels compared to NC. In iodine-treated cells, si-circ_0004851 reversed the iodine-induced changes in circ_0004851 and FGF11 expression and miR-296-3p reduction (Fig. 4B and C). Correspondingly, FGF11 protein expression mirrored mRNA trends. Proliferation assays indicated that si-circ_0004851 curtailed PTC cell proliferation, an effect inverted by high iodine concentrations (Fig. 5A). In invasion and wound healing assays, circ_0004851 silencing markedly reduced PTC cell migration and invasion, which high iodine levels counteracted (Fig. 5B and C). Flow cytometry showed that apoptosis was augmented (Fig. 5D), while elevated circ_0004851 levels enhanced proliferation, migration, invasion, as well as curbed apoptosis within PTC cells. Collectively, these outcomes implicate circ_0004851 in the regulation of PTC cell proliferation and survival in vitro. Given the role of circ_0004851 as an effective miR-296-3p sponge, we concentrated on exploring its target gene interactions.

### Mir-296-3p overexpression suppresses PTC Cell functions and induces apoptosis

**Fig. 6:**
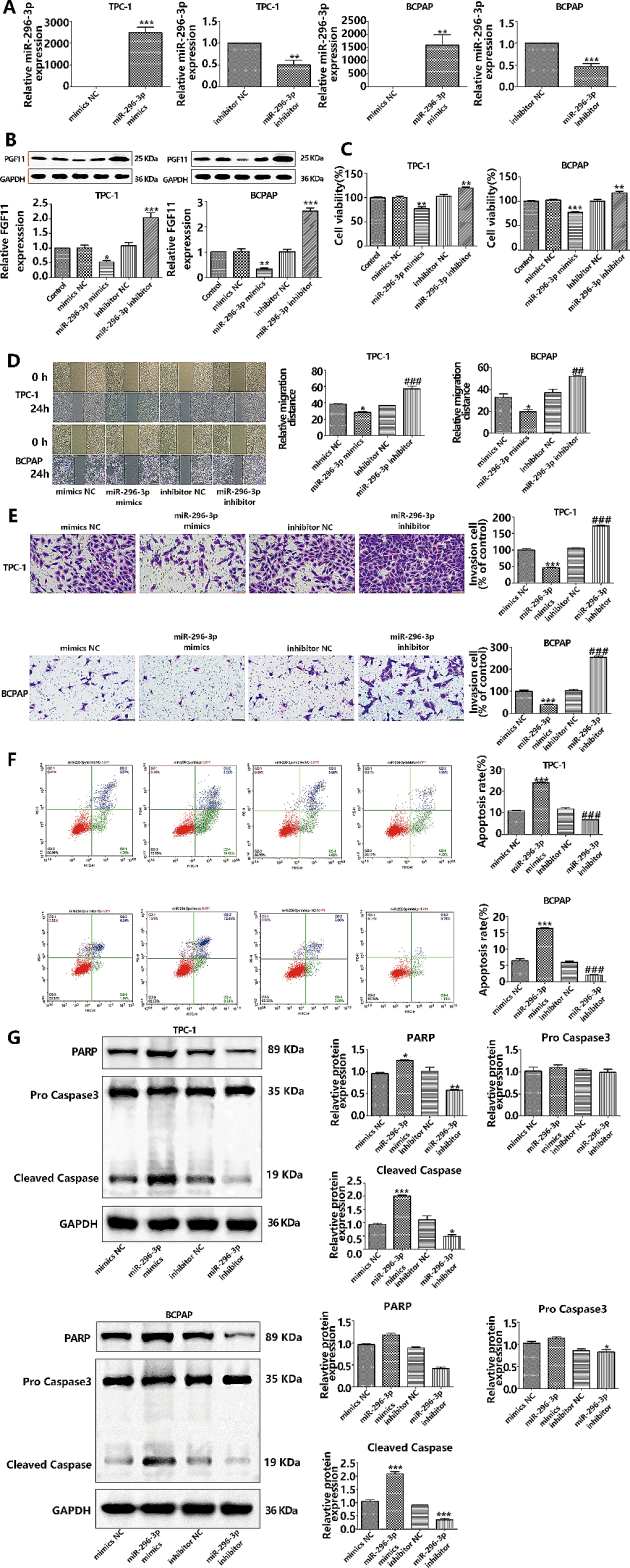
High expression of miR-296-3p inhibits the proliferation and invasion of PTC cells, promotes apoptosis. **A**.PCR was used to detect the transfection efficiency of miR-296-3p mimics or inhibitors in PTC cell lines; **B**. FGF11 protein levels in PTC cells were detected by Western blot analysis of miR-296-3p mimics, inhibitors and negative controls; **C.** The number of PTC cell lines that have been transfected with miR-296-3p mimics is dropping and inhibitors is increasing; **C**. The movement of PTC cell lines is affected by the transfection of miR-296-3p mimics or inhibitors; **D**. PTC cell lines that have been transfected with miR-296-3p mimics dropped and inhibitors increased invasion capabilities; **F**. PTC cell lines that have been transfected with miR-296-3p mimics increase and inhibitors undergo apoptosis. **G**. Western blot also showed that overexpression of miR-296-3p increased apoptosis-related protein cleaved caspase-3

The multifaceted role of miR-296-3p in various cancers is documented, but its impact on PTC is underexplored. Given circ_0004851’s sequestration of miR-296-3p, we investigated miR-296-3p’s function in PTC by transfecting TPC-1 and BCPAP cells with miR-296-3p mimics or inhibitors, with qRT-PCR verifying transfection efficiency (Fig. 6A). Western blot assessment monitored FGF11 protein levels (Fig. 6B), revealing no change in the control versus the NC group, thus indicating no impact from transfection reagents alone. miR-296-3p overexpression downregulated FGF11, while miR-296-3p inhibition upregulated it, confirming FGF11 as a direct target of miR-296-3p. CCK-8 assays demonstrated that elevated miR-296-3p levels reduced PTC cell proliferation, whereas miR-296-3p suppression led to elevated proliferation (Fig. 6C). Scratch and Transwell assays further established that miR-296-3p overexpression curtailed cell migration and invasion, with inhibition producing the opposite effect (Fig. 6D-E). Flow cytometry assessment showed that miR-296-3p upregulation augmented apoptosis in PTC cells, whereas downregulation reduced it (Fig. 6F). Western blot also showed that overexpression of miR-296-3p increased apoptosis-related proteins PARP and cleaved caspase-3 (Fig. 6G). Collectively, these findings indicate that miR-296-3p elevation suppresses PTC cell proliferation, migration, and invasion while promoting apoptosis.

### Silencing of mir-296-3p reverses the effects of si-circ_0004851 in PTC cells

**Fig. 7:**
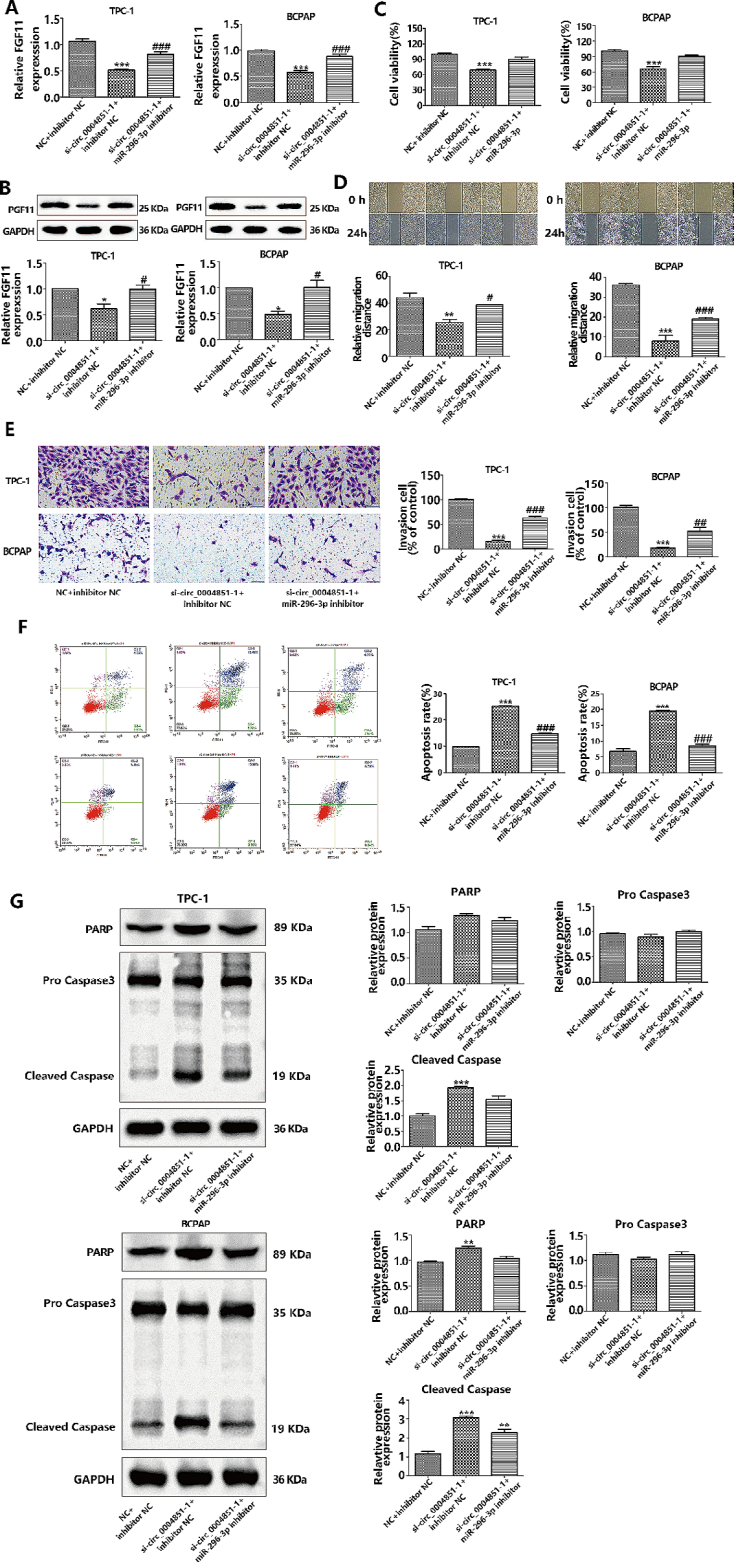
Silencing miR-296-3p reverses the effect of si-circ0004851 on PTC cells. **A**. mRNA expression of miR-296-3p inhibitor FGF11 in si-circ_0004851 or si-NC PTC cells with or without transfection; **B**. si-circ_0004851 or si-NC PTC cells transfected or did not transfect miR-296-3p inhibitor FGF11 protein levels; **C**. CCK-8 detection showed that circ_0004851 could eliminate the inhibition of cell growth by down-regulating miR-296-3p; **C.** Assessment of the ability of cell scratches to migrate; **D**. Assessment of invasive capacity through the Transwell test; **F**. Apoptotic capacity was detected by flow cytometry; **G.** The expressions of apoptosis-related proteins were detected by Western blot

Considering our hypothesis that circ_0004851 primarily modulates PTC progression by sequestering miR-296-3p, it was crucial to investigate whether miR-296-3p could counteract the impact of si-circ_0004851 on PTC cells. We conducted rescue experiments in TPC-1 and BCPAP cells co-transfected with si-circ_0004851 and si-NC, using a miR-296-3p inhibitor or control vector. qRT-PCR and western blot analyses demonstrated (Fig. 7A-B) that circ_0004851 knockdown in PTC cells resulted in the inhibition of FGF11 expression, while the miR-296-3p inhibitor reversed the inhibitory effect of si-circ_0004851 on FGF11. CCK-8 assessment revealed that exogenous down-regulation of miR-296-3p expression counteracted the growth inhibitory effect of si-circ_0004851 (Fig. 7C). In comparison to the NC group, miR-296-3p inhibition and si-circ_0004851 co-transfection partially rescued the impaired motility of si-circ_0004851 in PTC cell lines, as evidenced by cell scratch migration assays and Transwell invasion assays (Fig. 7C-D). Furthermore, apoptosis assays confirmed that miR-296-3p inhibition, along with si-circ_0004851, mitigated the apoptotic effect of si-circ_0004851 in PTC cells (Fig. 7F). Western blot also showed that miR-296-3p inhibition reversed the up-regulation of si-circ_0004851 on apoptosis-related proteins in PTC cells (Fig. 7G).

### Circ_0004851’s impact on PTC progression through FGF11

**Fig. 8:**
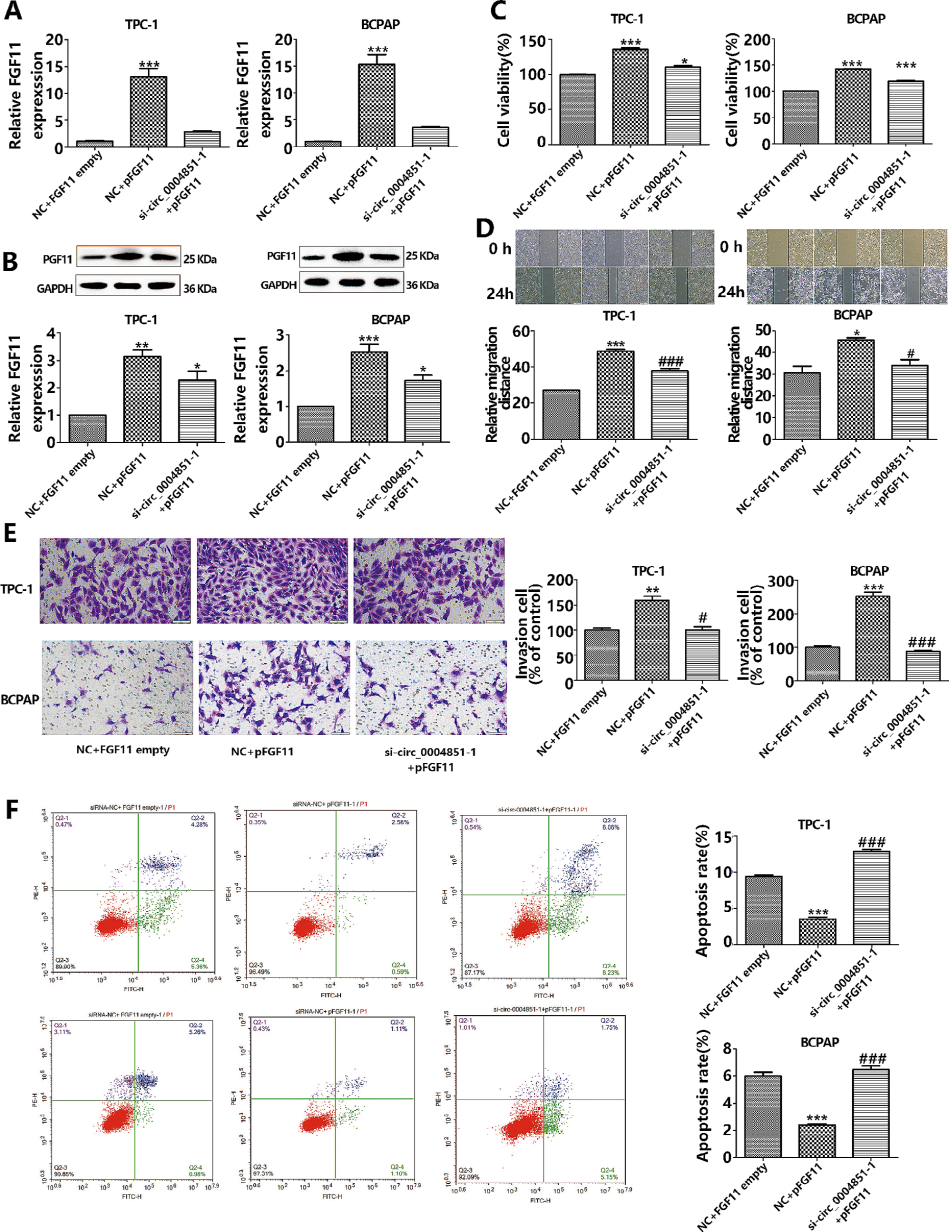
Circ0004851 affects PTC progress through FGF11. **A**. The expression of FGF11 mRNA in PTC cells co-transfected with si-NC + empty vector, si-NC + FGF11 over-expressing plasmid and si-Circ_0004851 + FGF11 over-expressing plasmid was detected by qRT-PCR; **B**. Western blot detection of FGF11 protein levels in PTC cells; **C.** CCK-8 assay showed that overexpression of FGF11 could eliminate the effect of Circ-004851 silencing on cell growth; **D.** Cell scratch detection of the effect of FGF11 overexpression on migration ability; **E.** Transwell invasion assay to detect the effect of FGF11 overexpression on invasion ability; **F**. Apoptosis was detected by flow cytometry

Although FGF11 has been identified as an oncogene in a number of cancer types, its function in PTC is unknown. In order to elucidate the association involving endogenous FGF11 expression and PTC pathogenesis, we generated an FGF11 overexpression plasmid and transfected it into circ_0004851-silenced TPC-1 and BCPAP cells. This aimed to investigate whether circ_0004851 influences PTC progression by targeting FGF11. We assessed proliferative, migratory, invasive, and apoptotic capabilities to delve into the effects of FGF11 overexpression on circ_0004851-deficient PTC cells. qRT-PCR and Western blot analyses demonstrated that the FGF11 overexpression plasmid significantly upregulated FGF11 expression. Furthermore, the overexpression of FGF11 upregulated circ_0004851-deficient cellular FGF11 expression (Fig. 8A-B). In addition, we observed that the proliferation, migration, and invasion abilities of cells, as assessed by cck-8, cell scratch, and Transwell assays, were enhanced upon FGF11 overexpression, while apoptosis was inhibited when compared to cells treated with the NC group. Moreover, overexpression of FGF11 in circ_0004851-deficient cells mitigated the inhibitory effects of si-circ_0004851 on proliferation, migration, invasion, and promoted apoptotic effects (Fig. 8C-F). Collectively, these findings suggest that circ_0004851 primarily contributes to PTC progression by targeting FGF11.

### Establishment of a nude mouse model to investigate high iodine’s promotion of PTC cell growth in vitro

**Fig. 9:**
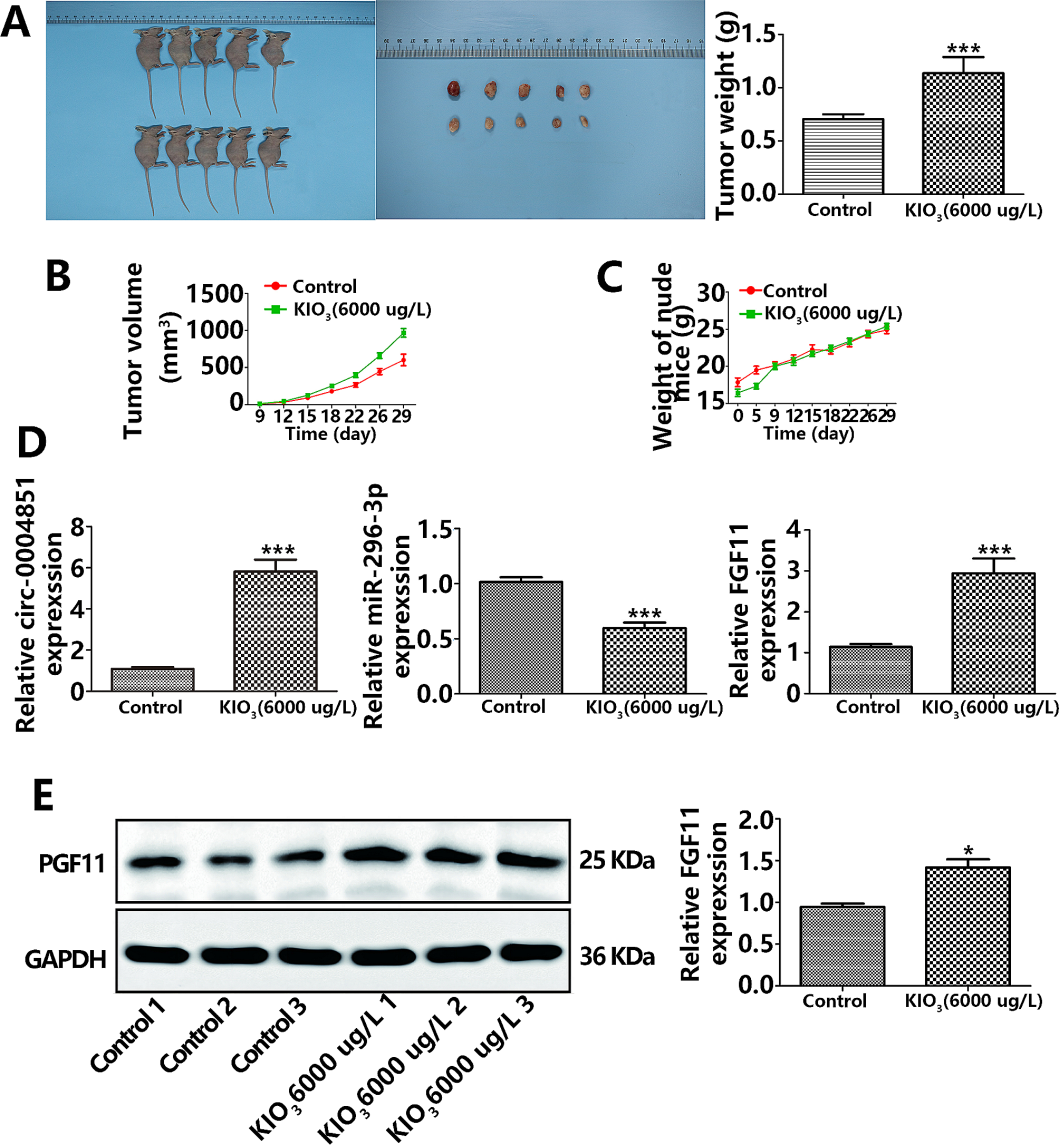
High iodine promoted tumor growth in xenograft tumor models of PTC cells. **A**. BCPAP cells were subcutaneously injected into nude mice under exposure to high iodine and tumor weights were measured at the end of the day 29; **B-C**. Relative tumor volumes and mice weight of different groups monitored every 3 days during treatment with or without high iodine; **D**. PCR for circ_0004851、miR-296-3p、FGF11 in xenograft tumor models; **E.** Western blot for FGF11 in xenograft tumor models. *p < 0.05, **p < 0.01, ***p < 0.001

Elevated levels of iodine are strongly implicated in thyroid cancer development [20]. To elucidate the role of high iodine in PTC progression, we established a xenograft tumor model. As depicted in Fig. 9A-B, the tumor volume and weight of mice in the high-iodine group exceeded those in the control group (p less than 0.05). Notably, the mice’s weight did not significantly differ between the two groups throughout the experiment (Fig. 9C). Furthermore, PCR and Western blot outcomes revealed that circ_0004851 expression elevated, while miR-296-3p expression reduced in the high iodine group. Additionally, FGF11 mRNA and protein expression levels were elevated in the high iodine group (Fig. 9D-E). These outcomes indicate that high iodine promotes tumor growth in the xenograft tumor model and significantly influences the expression of circ_0004851/miR-296-3p/FGF11 in this model.

## Discussion

**Fig. 10:**
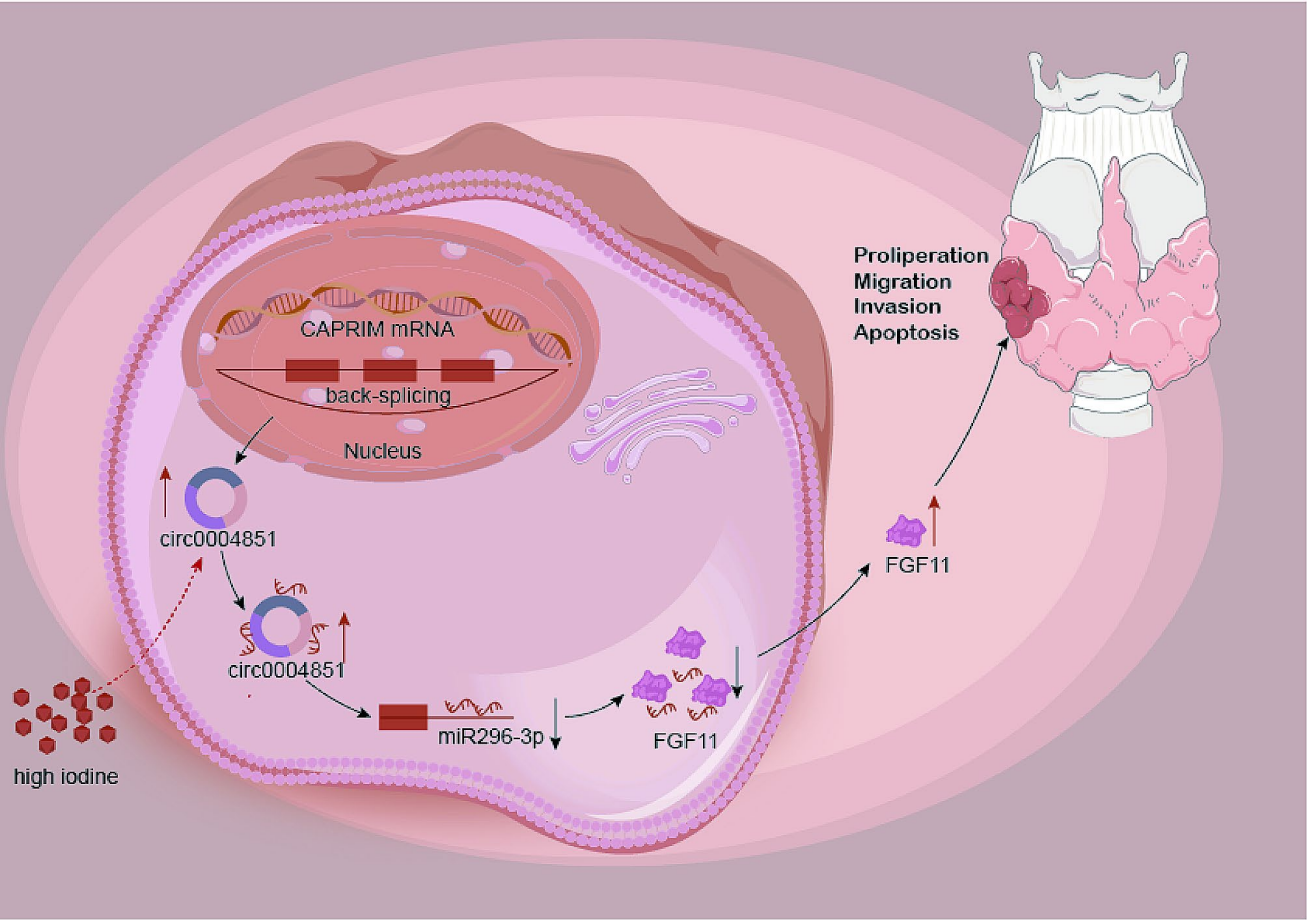
Schematic diagram of the mechanism by which hyperiodine regulates PTC cell proliferation, migration, apoptosis and invasion through circ_0004851/miR-296-3p/FGF11

Iodine is vital for life and represents the most universally essential element for all living organisms. However, excessive iodine intake can have adverse health consequences. Studies have demonstrated that excess iodine can lead to benign thyroid disorders, including autoimmune thyroiditis, benign thyroid nodules, hypothyroidism, and hyperthyroidism [21]. Paradoxically, elevated iodine intake appears to result in less aggressive thyroid cancer, particularly PTC [22]. Guan H et al. investigated the relationship between iodine intake and *BRAF* mutations in PTC and showed that the prevalence of *BRAF* mutations in PTC patients with high iodine intake was 69%, which was higher than the patients with normal iodine intake [23]. A Korean study found that both relatively excessive iodine intake and low iodine intake appear to be important risk factors for *BRAF* mutations in PTC patients [24]. A recent Meta-Analysis study found that the incidence of *BRAF* V600E mutations in PTC patients with high iodine intake was 77.6%, which was higher than 64.61% in patients with low iodine intake and 60.15% in patients with normal iodine intake [7]. Despite a considerable body of research on the impact of iodine on thyroid cancer tumorigenesis and progression, the association involving high iodine intake and TC risk remains contentious, and the mechanisms behind iodine excess in the development of PTC are still unclear [25–34]. Serum iodine levels reflect the actual iodine content in the body, offering a more accurate assessment of iodine nutritional status. Unlike urinary iodine, serum iodine is more stable and less susceptible to dietary fluctuations. Therefore, we opted to utilize blood iodine levels as a basis for evaluating the iodine nutritional status of the subjects in our research. Through clinical data assessment and a series of in vitro as well as in vivo experiments, we have established that elevated iodine levels contribute to the development of PTC. Furthermore, we identified the involvement of the circ_0004851/miR-296-3p/FGF11 axis in mediating the effects of iodine on PTC in our in vitro mechanistic investigation (Fig. 10).

In our research population, elevated blood iodine levels emerge as a risk factor for PTC. Our findings indicate that high blood iodine levels correlate with various aspects of PTC, including tumor location, size, number, and metastasis. Patients with elevated blood iodine values tend to present with enlarged and multiple tumors. This conclusion aligns with the outcomes of studies, such as one assessing serum iodine concentration, which classifies high serum iodine levels as a risk factor for thyroid diseases [35]. Another research suggests that high iodine intake could be linked to the growth of PTC rather than its initiation [31]. A meta-assessment of 22 cross-sectional studies conducted by Yan et al. indicates that high iodine intake does not pose a risk for PTC development and that high urinary iodine is a specific characteristic of the disease [36]. Some studies have identified associations between excessive iodine intake and elevated risks of lymph node metastasis, larger tumors, peritumoral invasion, bilateral tumor location, and extra-thyroidal metastases. However, others have failed to establish such associations regarding lymph node metastasis, tumor size, multifocal tumors, or bilateral and extra-peritumoral extension [5, 37–40]. The inconsistency in previous studies’ findings on the association involving dietary iodine intake as well as thyroid cancer outcomes from variations in measurement methods and potential measurement biases associated with assessing iodine intake [41, 42].

In our research, we observed that iodine at large concentrations inhibited apoptosis while promoting proliferation, migration, and invasion of PTC cells. Previous research reported that TPC-1 cells containing 100 μm Ki (high iodine) exhibited significantly elevated proliferation and migration compared to a cell line containing 25 μm Ki [15]. Additionally, KIO_3_ was found to have a tumor-promoting effect (elevated migration and invasion) on SW579 (a human thyroid cancer cell line) cells at a concentration of 10^− 6^ mol/L, aligning with our research’s outcomes [17]. Our mouse experiments corroborate the findings of Lv et al. [43] that exposure to 2 mmol/L KIO_3_ for 72 h elevated the proliferation of thyroid cancer cells (BCPAP and 8305 C) and that high iodine promoted tumor growth in a mouse transplantation tumor model. elevated iodine concentrations promote the proliferation of PTC cells, as indicated by a heightened rate of proliferation [44]. Therefore, it is reasonable to infer that elevated iodine intake exerts a significant influence on PTC development.

Our research identified dysregulation of numerous circRNAs, miRNAs, and mRNAs in PTC tissues from patients exposed to high iodine. Some of these miRNAs and mRNAs have previously been implicated in the mechanism of PTC progression under high iodine conditions [45]. Consequently, we postulate that the differentially expressed genes identified through RNA sequencing in iodine-exposed PTC tissues could play a role in the progression of PTC tissues.

CircRNAs play a significant functional role in tumorigenesis [46, 47]. Although circRNA research is still in its early stages and their roles in PTC occurrence and progression are not well-understood, a growing number of novel circRNAs have been identified that could have oncogenic roles in thyroid cancer [48]. In addition to direct protein interactions, circRNAs contribute to TC progression by acting as miRNA sponges. For instance, the overexpression of circ-PSD3 sponges miR-7-5p in PTC, reducing the inhibition of METTL7B by miR-7-5p, thereby promoting the proliferation and invasion of PTC cells [49]. Similarly, circular RNA circRUNX1 promotes PTC progression and metastasis by sponging miR-296-3p and regulating DDHD2 expression [50]. Circ-0001018, circ-0006156, circ_0058124, and circ_0062389 have been implicated in the regulation of various signaling pathways associated with TC cell proliferation and apoptosis [51–54]. In our research, we investigated iodine’s role in PTC progression through the circ_0004851-associated ceRNA network (circ_0004851/miR-296-3p/FGF11 axis) using RNA sequencing, which we subsequently validated in PTC tissues, cells, and xenograft tumor mice. We focused on a specific circRNA, circ-0004851, which plays a pivotal role in PTC. We observed high expression levels of circ_0004851 in both PTC tissues and cells, consistent with our high-throughput sequencing outcomes for PTC tissues. This led us to hypothesize that circ_0004851 could play a crucial role in PTC progression. Initially, we elucidated the structure of circ_0004851 in PTC. Following the down-regulation of circ_0004851 through RNA interference, we observed reduced proliferation, invasion, and migration in PTC cells, accompanied by elevated apoptosis. Circ-0004851 was abundantly expressed in PTC under high iodine exposure, contributing to worse biological behavior at elevated levels. These findings suggest that changes in PTC cell behavior due to high iodine are associated with regulated circ_0004851 levels, highlighting its pivotal role in PTC progression. In our research, we found that miR-296-3p exhibited a strong binding affinity to both circ-0004851 and FGF11, a relationship confirmed through bioinformatics, qPCR, luciferase, and FISH analyses. This underscores the significant role of miR-296-3p in thyroid tumor progression and metastasis. Mechanistic studies further revealed that a miR-296-3p inhibitor reversed the downregulation of FGF11 expression induced by circ-0004851 knockdown in PTC cells, along with the inhibition of PTC progression in vitro. These outcomes establish a novel association between miR-296-3p, circ-0004851, and the regulation of PTC. Circ-0004851 acts as a sponge for miR-296-3p and miR-296-3p as a critical negative regulator of FGF11, suggesting a network regulatory relationship among circ-0004851, miR-296-3p, and FGF11 in PTC. FGF11 was significantly upregulated in PTC under high iodine exposure, and its overexpression promoted tumor cell proliferation, invasion, migration, and inhibited apoptosis. This indicates a close connection between FGF11 and PTC development and biological characteristics. FGF11 expression, a target of miR-296-3p, was positively regulated by circ-0004851 in rescue experiments, and the suppression of circ-0004851 mitigated the tumor-promoting effects of overexpressed FGF11. The outcomes underscore the function of FGF11 to promote PTC cell progression and further validate circ-0004851 as a ceRNA for miR-296-3p, providing valuable evidence for the involvement of circ-0004851 in PTC development. These findings unveil an unreported correlation between FGF11, miR-296-3p, and circ-0004851, culminating in the identification of a mechanism where circ-0004851 functions as a miR-296-3p sponge to drive PTC development.

Hypoxia is a significant cause of metastasis and recurrence in cervical lymph nodes of PTC, and mechanistic studies reveal that in a hypoxic environment, FGF11 forms a positive feedback loop with HIF1α, which in turn promotes thyroid cancer growth and metastasis [55]. However, no study has yet demonstrated the positive feedback loop between circ-0004851 and miR-296-3p has a regulatory relationship with HIF1α. A study has identified the role of miR-296-3p in targeting ether-à-go-go (EAG1) to regulate cell growth in human glioblastoma [56], while EAG1 can promote tumor angiogenesis by increasing HIF1 activity [57]. In Chinese hamster ovary cells and mouse embryonic fibroblasts, HIF-1α levels were similarly elevated by EAG1 expression [58]. In addition, in non-small cell lung cancer, miR-296-3p acts as a tumor suppressor and inhibits tumor cell migration and invasion by targeting apurinic/apyrimidinic endodeoxyribonuclease 1 (APEX1) expression [59], and APEX1 can further promote the transcriptional activation of HIF1 or hypoxia-inducible factor-like factor (HLF) as a hypoxia-related gene [60]. The above findings suggest that miR-296-3p may indirectly regulate the level of HIF1α by targeting and regulating its downstream genes.

Iodine intake may induce *BRAF* V600E gene mutations in PTC patients through various indirect pathways [7]. However, the effect of *BRAF* V600E gene mutation on the circ-0004851/miR-296-3p/FGF11 pathway is unknown. Previous studies have found that the *BRAF* V600E mutation in PTC is associated with glucose transporter 1 (GLUT1) overexpression, which may contribute to the initiation of a glycolytic phenotype in cancer cells [61]. The YAP/HIF-1α complex binds to the GLUT1 gene and directly activates its transcription, which accelerates the PTC cells’ glycolysis under hypoxic conditions [62]. The above findings suggest that BRAF mutations may enhance the circ_0004851/miR-296-3p/FGF11 pathway by affecting the binding of GLUT1 to the YAP/HIF-1α complex.

In this research, we included a substantial number of clinical cases, enabling a comprehensive exploration of the association involving high iodine as well as the clinical characteristics of PTC patients. Our investigation extended to the cellular level, where we validated that iodine’s effects on various biological behaviors align with the clinical data. Leveraging the advantages of high-throughput sequencing, such as speed, accuracy, and efficiency, we delved into the molecular mechanism through which iodine influences PTC. Following repeated interventions on upstream and downstream genes and thorough validation of their effects on biological behaviors, we established the mechanism by which iodine affects PTC. However, it’s important to acknowledge the limitations of this research. Determination of circulating serum iodine in patients is a tricky issue, and iodine levels are dependent on the patient’s living environment and diet. In the present study, we did not assess serum iodine levels in patients before the onset of PTC, nor did we continuously observe the dynamics of serum iodine levels, which may affect some of the conclusions about the relationship between their levels and tumor characteristics. Similarly, we assessed the impact of various concentrations of KIO_3_ on normal thyroid cells and malignant PTC cells, yet we did not investigate the time-dependent effects of KIO_3_ across various cell lines at various time points. This aspect should be considered in our future research. Another limitation pertains to our classification; we focused solely on papillary thyroid carcinoma, failing to examine multiple thyroid cancer subtypes and explore the effects and molecular mechanisms of high iodine on PTC in these subtypes. Furthermore, the critical next step involves conducting live animal experiments to intervene with circ_0004851/miR-296-3p/FGF11 and validate its molecular mechanism. Finally, the translational significance of our findings in the clinical context requires further confirmation.

## Conclusion

In summary, our data confirm that high iodine regulates tumor progression in PTC through the circ_0004851/miR-296-3p/FGF11 pathway. With the global focus on iodine and thyroid cancer, our findings provide further evidence for the role of iodine in PTC and enrich the research of non-coding RNAs in iodine-related thyroid cancer. Additionally, we suggest that targeting the circ_0004851/miR-296-3p/FGF11 axis is a potential strategy for the treatment of PTC, and that circRNA silencing through the ceRNA mechanism could play a crucial therapeutic role in resisting high iodine-promoted tumor progression.

### Electronic supplementary material

Below is the link to the electronic supplementary material.

Supplementary Material 1

Supplementary Material 2

## Data Availability

As the initial contributions detailed in the research are presently undergoing additional evaluation in preparation for their submission to additional funding initiatives, they have not yet been made available in public databases. Please contact the corresponding author with the applicable justifications if necessary. We shall disclose our data provided that no conflicts arise.
