# Supplementary material for: Circ_0004851 regulates the molecular mechanism of miR-296-3p/FGF11 in the influence of high iodine on PTC
Source: J Transl Med. 2024 Jun 20;22:586. doi: 10.1186/s12967-024-05405-2 (PMC11191183; doi:10.1186/s12967-024-05405-2)
Supplement: Supplementary file 1 — Supplementary Material 1 [file 12967_2024_5405_MOESM1_ESM.docx]

**Table S1. Primer sequences**

| Gene | Primer sequences |
| --- | --- |
| FN1-F | GGGAGAAGTATGTGCATGGTGT |
| FN1-R | GACAGGACCACTTGAGCTTGG |
| TNXB-F | TCCTCACCACGGTTCCTGAC |
| TNXB-R | ACGTCATAGGTGTCCACAGGA |
| FGF11-F | TACCAGCTCCTTCACCCACTTC |
| FGF11-R | GCGAACTGTAGAGCAGTCCCT |
| SOD3-F | CTTGGAGGAGCTGGAAAGGTGC |
| SOD3-R | ACATGTCTCGGATCCACTCCGC |
| LMOD1-F | ACCAGACGGAGAAACAGTCCA |
| LMOD1-R | CTCCACTTGCTTGCTTTCATCCA |
| CLSTN2-F | TGCCCAAGAACCTGACCGAT |
| CLSTN2-R | TGATGCCGGTTCATTTCGGTT |
| hsa_Circ_0073486-F | GCTCCAGTTCATATCACGGCA |
| hsa_Circ_0073486-R | TCCGAGTCTACCACAATGCTGA |
| hsa_Circ_0005769-F | GTGGCTTCTATGTTTTGGGTTGA |
| hsa_Circ_0005769-R | ATTCTCAATAGCTCTAAGGCCACT |
| hsa_Circ_0004851-F | AGCTTCAAACAGGTTCCTTTGGT |
| hsa_Circ_0004851-R | GTTCCTTCTGTGGTCGTTGCT |
| hsa-miR-204-3p（tissue） | TGGGAAGGCAAAGGGACGT |
| hsa-miR-146b-3p（tissue） | CCCTGTGGACTCAGTTCTGGT |
| hsa-miR-296-3p（tissue） | GAGGGTTGGGTGGAGGCTCTCC |
| hsa-miR-296-3p-F（cell） | CGGAGGGTTGGGTGGAGG |
| hsa-miR-296-3p-R（cell） | AGTGCAGGGTCCAGGTATT |
| hsa-miR-296-3p-RT（cell） | GTCGTATCCAGTGCAGGGTCCGAGGTATTCGCACTGGATACGACGGAGAG |
| GAPDH-F | GGTATCGTGGAAGGACTCATGAC |
| GAPDH-R | ATGCCAGTGAGCTTCCCGTTCAG |
| U6-F | CTCGCTTCGGCAGCACATATACT |
| U6-R | ACGCTTCACGAATTTGCGTGTC |
| U6-RT | AAAATATGGAACGCTTCACGAATTTG |
