# Supplementary material for: Circ_0004851 regulates the molecular mechanism of miR-296-3p/FGF11 in the influence of high iodine on PTC
Source: J Transl Med. 2024 Jun 20;22:586. doi: 10.1186/s12967-024-05405-2 (PMC11191183; doi:10.1186/s12967-024-05405-2)
Supplement: Supplementary file 2 — Supplementary Material 2 [file 12967_2024_5405_MOESM2_ESM.docx]

**Table S2. Sequences information**

| Gene | Sense | Antisense |
| --- | --- | --- |
| Negative control | 5’-UUC UCC GAA CGU GUC ACG UTT-3’ | 5’-ACG UGA CAC GUU CGG AGA ATT-3’ |
| hsa_circ_0004851-1 | AACAGCUUCAAACAGGUUCTT | GAACCUGUUUGAAGCUGUUTT |
| hsa_circ_0004851-2 | CAGCUUCAAACAGGUUCCUTT | AGGAACCUGUUUGAAGCUGTT |
| hsa-miR-296-3p mimics | GAGGGUUGGGUGGAGGCUCUCC | AGAGCCUCCACCCAACCCUCUU |
| mircoRNA inhibitor N.C. | CAGUACUUUUGUGUAGUACAA |  |
| hsa-miR-296-3p inhibitor | GGAGAGCCUCCACCCAACCCUC |  |
| mircoRNA inhibitor N.C. | CAGUACUUUUGUGUAGUACAA |  |
